# Painful Temporomandibular Disorders, Bruxism and Oral Parafunctions before and during the COVID-19 Pandemic Era: A Sex Comparison among Dental Patients

**DOI:** 10.3390/jcm11030589

**Published:** 2022-01-25

**Authors:** Orit Winocur-Arias, Efraim Winocur, Tamar Shalev-Antsel, Shoshana Reiter, Levartovsky Shifra, Alona Emodi-Perlman, Pessia Friedman-Rubin

**Affiliations:** 1 Department of Oral Pathology and Oral Medicine, The Maurice and Gabriela Goldschleger School of Dental Medicine, Tel Aviv University, Tel Aviv 6139001, Israel; orit_winocur@yahoo.com (O.W.-A.); shoshana.reiter@gmail.com (R.S.); 2 Department of Oral Rehabilitation, The Maurice and Gabriela Goldschleger School of Dental Medicine, Tel Aviv University, Tel Aviv 6139001, Israel; winocur@tauex.tau.ac.il (E.W.); tamarashalev@gmail.com (T.S.-A.); shifra@tauex.tau.ac.il (L.S.); pessia80@gmail.com (P.F.-R.)

**Keywords:** awake bruxism, sleep bruxism, oral parafunctions, painful temporomandibular disorders, COVID-19, gender

## Abstract

Aim: To evaluate the effect of the current coronavirus pandemic on the prevalence of bruxism, oral parafunctions and painful Temporo-Mandibular Disorders (TMDs) and to evaluate the influence of the pandemic on both sexes. Methods: This retrospective study included 288 dental patients who underwent complete anamnesis and examination according to the Diagnostic Criteria for TMD. The study evaluated two patient populations according to the date of examination: (a) pre-COVID-19 pandemic era (108 patients); (b) COVID 19 pandemic era, where 180 patients were examined during the pandemic. Results: A significant increase in parafunction activity was found in both men and women (*p* < 0.001) during the COVID-19 pandemic. Awake bruxism (AB) and sleep bruxism (SB) was more prevalent during the COVID-19 pandemic solely in women (AB-*p* < 0.001; SB-*p* = 0.014). Conclusions: Men and women were affected by the ongoing stress due to the COVID-19 pandemic, yet women showed a higher influence as compared to men. The long-term exposure to elevated levels of anxiety and stress may aggravate or trigger stomatognathic detrimental conditions. Dentists should be aware and regularly monitor their patients regarding the possible existence and consequences of bruxism and TMD.

## 1. Introduction

The definition of bruxism was set in 2013 by a panel of experts, stating that bruxism is considered a “repetitive jaw muscle activity characterized by clenching or grinding of the teeth and/or bracing or thrusting of the mandible” [1]. Bruxism has two distinct circadian manifestations: sleep bruxism and awake bruxism. In 2018, the same panel published an update consensus that developed separate definitions for sleep and awake bruxism. They concluded that sleep and awake bruxism are masticatory muscle activities that occur during sleep (characterized as rhythmic or non-rhythmic) and wakefulness (characterized by repetitive or sustained tooth contact and/or by bracing or thrusting of the mandible), respectively. They claimed that in otherwise healthy individuals bruxism should not be considered as a disorder but rather as a behavior that may be a risk (and/or protective) factor for certain clinical consequences [2]. From the existing literature on the etiology of bruxism, it seems that it results from a combination of several factors, including physiological/biological, psychosocial and exogenous factors [3,4,5,6,7,8,9,10]. Anxiety and stress sensitivity have been associated with bruxism [3,4,5,6]. SB declines over time, from 14% in children to 8% in adults, to 3% in patients over 60 years of age [11]. This is in contrast to oral lesions, which are more prominent in the mature and elderly age groups [12].

Oral parafunctions (OP) are non-physiological oral functions that do not serve any functional purpose and should be differentiated from physiological jaw activities (i.e., mastication, swallowing, talking) [13]. Both bruxism and OP are usually harmless and share a common stress sensitivity association in their etiology [14]. However, when the activity exceeds the individual’s physiologic tolerance, the stomatognathic system begins to alter and a breakdown may occur. The initial breakdown occurs in the tissue with the lowest structural tolerance and may act as a risk factor for the development of orofacial pain and temporomandibular disorders (TMDs) [15,16,17].

TMDs are a group of conditions that cause pain and dysfunction of the masticatory muscles, the temporomandibular joints (TMJs) and associated structures. The most common features of TMD are regional pain, limited jaw movements and acoustic sounds from TMJs during mandibular motions [18]. TMD patients often report that symptoms increase during stressful situations [19,20,21].

Both TMD and bruxism are prevalent in the adult general population, with more than 50% of the population presenting signs of TMDs [22], 22%–30% presenting AB and 8–15% SB [23].

The pandemic named COVID-19 (Coronavirus Disease 2019) caused by the SARS-CoV-2 (Severe Acute Respiratory Syndrome Coronavirus 2) infection was first detected in late December 2019 in Wuhan China and has spread since all over the globe. Until the manufacturing of a vaccine, the policy adopted by most countries was social distancing and partial-to-total lockdown. This is still the policy today, while a fifth wave is threatening to spread. This policy of social isolation, despite its effectiveness, dramatically altered everyday routine life of the world population, giving rise to severe existential, economic, social, mental and emotional health threats inducing stress, anxiety, and depression [24,25]. These psychosocial factors are notable stressors capable of causing bruxism activity (sleep and awake), oral parafunctions performance and TMDs. A previous online survey study performed on a general population during the first lockdown and conducted by members of this group [25] demonstrated that “the psycho emotional status caused by COVID-19 resulted in bruxism and TMD symptoms intensification”.

Accordingly, the study aim was two-fold: (i) This time, our group wanted to evaluate the effect of the current coronavirus pandemic on the prevalence of bruxism, oral parafunctions and TMDs in a general dental patient population; and (ii) evaluate the influence of the pandemic on both sexes.

The null hypothesis was that the coronavirus pandemic will have no influence on the prevalence of bruxism, oral parafunctions and TMDs and that no differences will be found between sexes.

## 2. Methods

### 2.1. Ethical Concerns

The study was approved by the Tel Aviv University Institutional Ethical Committee prior to data collection (ID: 0002338-1). Informed consent was waived, since the data were retrieved retrospectively and each patient who is referred to the student’s clinic routinely signs a form in which he/she agrees that his/her data may be anonymously used for research purposes. The study was self-funded by the authors.

### 2.2. Study Population

This retrospective study included 311 patients who underwent complete anamnesis and examination at the Maurice and Gabriela Goldschleger Tel Aviv University student’s clinic from October 2018 to June 2021. Patients with incomplete anamnesis were not included in the database. Patient age originally ranged between 16–79. Patients under 18 were excluded (since the reliability of the DC/TMD has been tested only on the adult population), and patients over 65 were also excluded (to avoid the bias of systemic involvement and the fact that the inflammatory-degenerative joint disorders diagnosis is the most prominent in this group of age [26]. This diagnosis is not discussed in the present study). In addition, patients suffering from neurological disturbances, uncontrolled hormonal disease, neoplasm, psychiatric problems, history of facial or cervical injury, patients under certain medication (anti-depressants, myorelaxants) and drug users were excluded from the database. The final study population comprised 288 patients (mean age 35.6, SD 12.4) including: 155 men (mean age 33.9 SD 11) and 133 women (37.6 SD 13.6).

Two different groups of patients were evaluated:

1. Pre-COVID-19 pandemic: This group consisted of patients examined before the pandemic era (October 2018–February 2020).
2. COVID-19 pandemic: This group consisted of patients examined during the pandemic era (March 2020–June 2021)

### 2.3. Clinical Examination

The following clinical examination was part of the routine in the obligatory comprehensive examination before the student was allowed to begin dental treatment of the patient.

1. Temporomandibular examination and diagnosis: All patients were diagnosed according to the Axis I of the Diagnostic Criteria for Temporomandibular Disorders (DC/TMD) by a student. The accuracy of the examination was verified by a senior staff instructor who was officially calibrated and certified in the DC/TMD Training and Calibration Course at the Department of Orofacial Pain and Jaw function at the Faculty of Odontology at Malmö University, Sweden. Diagnosis of TMD was based on the criteria of the international DC/TMD consortium (http://rdc-tmdinternational.or). In order to obtain a final TMD diagnosis the patients were requested to fill in the official Hebrew version of the Symptoms Questionnaire [27].
2. Sleep & Awake Bruxism (AB & SB): The diagnosis of SB and AB depended upon the respondent’s awareness according to the Oral Behavior Checklist official Hebrew version [27]. It was recently recommended that the diagnosis of bruxism should be graded “possible” (when based solely on self-reports), “probable” (when based on clinical examination), or “definite” (when based on a polysomnographic recording with a simultaneous audio-video recording) [2]. Accordingly, the SB and AB diagnosis in this study should be graded as “possible”.
3. Parafunctional activity: The following oral habits were investigated: only patients who answered that they perform the habit at least 1–3 times a week were recorded as positive
  - Biting down on hard objects (pens, pencils, etc.);
  - Crushing hard candies, ice, popsicles, etc., with the teeth;
  - Biting fingernails;
  - Opening bottles with the teeth;
  - Gum chewing—average number of hours per day;
  - “Jaw-play” (involuntary small mandibular movements without tooth contact).

The final analysis included the following:

1. Painful TMD was defined by the presence of at least one of the following: myalgia, myofascial pain, headache attributed to TMD or arthralgia;
2. Possible sleep bruxism (SB);
3. Possible awake bruxism (AB);
4. Parafunctional Activity: Patients who reported performing at least one parafunction at least 1–3 times a week were recorded as positive;
5. Reported data from the official Hebrew version of the Symptoms Questionnaire [27].

### 2.4. Statistical Methods

The data were analyzed using IBM SPSS statistics version 25.0. (SPSS, Inc., Chicago, IL, USA). The level of statistical significance was assumed at *p* = 0.05, i.e., the results of statistical tests that appeared with a probability of *p* < 0.05 were considered statistically significant. Categoric variables were described as frequency and percentage. A Pearson Chi square test followed by Fishers’ Exact Test were used to test the association between those categorial variables. The independent variables for analysis included: Parafunction, Pain in jaw function, Pain on awakening, Reported TMD pain, Pain on Temporal area, Sleep bruxism, Awake bruxism, and Painful TMD. All tests were 2-tailed. A proportion test with Bonferroni correction was applied as appropriate.

## 3. Results

A total of 311 patients who underwent complete anamnesis and examination at the Maurice and Gabriela Goldschleger Tel Aviv University student’s clinic from 10/2018 to 6/2021 were included in the database. After exclusions, the final study population comprised 288 patients (mean age 35.6, SD 12.4) 155 men (mean age 33.9, SD 11) and 133 women (mean age 37.6, SD13.6).

All patients underwent complete anamnesis as detailed. In the first stage the results were compared between men and women regardless to the time of examination. After Bonferroni correction a significant difference between men and women was only found in reported TMD pain (*p* = 0.042) (Table 1). Painful TMD diagnosis was significantly more prevalent in women (*p* = 0.046).

Two different groups of patients were evaluated:

1. Pre-COVID 19 pandemic: This group consisted of 108 patients examined before the pandemic era (10/18–2/20).
2. COVID 19 pandemic: This group consisted of 180 patients examined during the pandemic era (3/20–6/21).

No significant differences were found between the groups regarding sex and age (Table 2).

After Bonferroni correction, a significant increase in the parafunction activity was found in both men and women during the COVID-19 pandemic era (*p* < 0.001). AB and SB remained significantly more prevalent during the COVID-19 pandemic only in women (*p* < 0.001 and 0.014 accordingly).

Although pain-in-jaw function and AB in men showed an increase during the COVID-19, following Bonferroni correction the significance was lost. No significance was found in reported TMD pain, pain on awakening and pain in the temporal area in both sexes even before the Bonferroni correction (Table 3).

Regarding patient reporting the performance of both SB and AB (SB & AB) a statistically significant prevalence increase was found in both sexes during the COVID-19 pandemic. The significance level was higher in women (*p* = 0.036 in men vs. *p* < 0.001 in women) (Table 4).

No significant difference in the prevalence of painful TMD diagnosis (according to the DC/TMD) was found between the two periods under study in both men and women.

## 4. Discussion

The present study aimed to investigate whether in a general patient population the COVID-19 pandemic influenced the prevalence of bruxism behaviors (AB and SB), oral parafunctional activity (OP) and painful TMDs. Another interesting issue was whether men and women reacted differently.

The null hypothesis of the present study was rejected since the Coronavirus pandemic was found to influence the prevalence of bruxism, oral parafunctions and TMDs. Differences were found between sexes.

It was found that oral parafunction activity was significantly more prevalent during the COVID-19 pandemic era in both men and women, while awake bruxism, after Bonferroni correction, remained significant only for women (*p* < 0.001). Previous studies, mostly self- report surveys [23,28,29,30,31], showed a possible association between stress and anxiety caused by the pandemic as well as an increase in self-reported bruxism behaviors. Generally, women seemed to suffer more from stressors such as: employment loss, increase in household chores and childcare burden [31,32,33] than men. It was also found that the rate of women who reported feelings of loneliness, stress and anxiety and fear of contracting the virus was higher than that of men [34]. Evidence to this is the fact that women were the majority of callers for emotional support via telephone (68% as compared to 32% men) [35]. These findings may explain the results found in the study.

The increase in awake masticatory muscle activity (AB) and OP during the pandemic years may further be explained by the adaptive stress coping theory [36]. According to this theory bruxism may play a role in stress reduction by releasing cortisol and producing salivary chromogranin, which reduces the negative mood [37,38]. Soto et al. in their study [36] demonstrated that “awake bruxer participants showed higher levels of adaptive coping strategies such as positive reappraisal”, implying that awake oral behaviors may play a protective role in an ongoing stressful situation, such as the one caused by the pandemic. Muscle contraction may be part of the defense behavior fight or flight responses, associated with stressful episodes [38,39,40,41].

Another adverse effect of social isolation, quarantine, confinement, financial instability and continuous stress and anxiety is poor sleep quality. In an online survey conducted in Italy during the early stages of the pandemic, more than half of the participants reported an increase in difficulty falling asleep and disturbed and/or restless sleep [28]. Such changes in sleep routine were also reported among 72% of university students in Brazil [29]. Polysomnographic studies show that SB is associated with transient arousal response, poor sleep quality and altered sleep architecture [42]. The present study found an increased prevalence of SB in women solely (*p* = 0.014 after Bonferroni correction). This is in accordance with Bigalke et al. [43], who found that women reported higher prevalence of increased general anxiety due to COVID-19 and reduced sleep quality compared to men. This may explain the significant increase in SB found in women during the pandemic years compared to men. Another possible explanation may be the increase in report of unhealthy exogenous SB risk factors [44,45] such as alcohol consumption and tobacco smoking during the pandemic [46,47].

In the present study, a group of patients reporting the performance of both SB and AB was detected. This group exhibited a statistically significant prevalence increase during the pandemic era in both sexes. The significance level was higher in women (*p* < 0.001 in women vs. *p* = 0.036 in men). The literature supports the fact that a certain number of individuals will report both behaviors (SB and AB) [48,49] and that they may present a more neurotic trait of personality with a higher stress sensitivity and anxious expectations [50]. This may explain the increase in prevalence observed in this group of patients during the pandemic era.

In the present study, painful TMD diagnosis was significantly more prevalent in women than in men (*p* = 0.046). These findings are in accordance with previous studies performed on a general population [51,52,53,54] and are well documented in the TMD literature. Surprisingly, despite the increase in prevalence of AB and OP which are recognized as risk factors for the development of TMDs, painful TMD prevalence was not significantly different between the two periods under study. The causal connection between AB, OP and the development of TMDs may be true in the presence of maladaptive coping strategies or the absence of positive ones. In these conditions increase in perceived stress may provoke TMDs [55]. As the study population was not a TMD clinic population it is possible that in this general patient population AB played a positive psychological role, allowing discharge of the psychological tension [37,38]. This finding is in line with a recent systematic review indicating that “the current scientific literature does not support a direct linear causal relationship between bruxism and musculoskeletal signs/symptoms” [56]. This indicates a more complex relationship, depending on the presence of other risk factors [57] rather than only on the amount of the masticatory muscle activity performed. It is also logical to assume that since the cohort was comprised of a dental clinic population, patients suffering from painful TMD refrained from attending the clinic during the pandemic period, causing a selection bias. This is one limitation of the study. Another limitation lies in the fact that although the TMD examination was carried out according to the DC/TMD axis Ino axis II instruments assessing stress, depression and anxiety were used. A last limitation was that smoking status and possible other bias factors were not assessed.

## 5. Conclusions

Both men and women were affected by the COVID-19 pandemic, yet the impact on women was stronger. The ongoing stress due to the COVID-19 pandemic has already caused aggravation of psychological and emotional distress, sleep disorders and an increase in unhealthy behavior among many individuals [58,59,60,61]. All these endogenous and exogenous factors may explain the increase observed in AB, SB, OP and TMDs. As we are entering the third year of the pandemic, the long-term exposure to elevated levels of anxiety and stress and the resulting increase in masticatory muscle activity may show different non-reversible physiological and psychological conditions over time. This may aggravate or trigger stomatognathic conditions. Caregivers and dentists in particular should be aware and regularly monitor their patients for the possible existence and consequences of AB, OP, SB and TMDs especially in stressful times such as a pandemic, in order to prevent the possible harmful consequences of such behavior over time. Dentists should also keep in mind that it is possible that periodontal status may indicate the risk and the severity of COVID-19 [62]. In an era of personalized medicine, dentists should be aware of the different risk factors among the sexes and discuss them with their patients.

## Figures and Tables

**Table 1:** Sex comparison regardless time of examination.

	Men	Women	*p*^
Parafunction	78 (50.3%)	62 (46.6%)	NS
Pain in jaw function	15 (9.7%)	26 (19.5%)	NS
Pain on awakening	17 (11.1%)	16 (12%)	NS
Reported TMD pain	27 (17.5%)	41(30.8%)	0.042
Pain on Temporal area	25 (16.3%)	32 (24.1%)	NS
Sleep bruxism	36 (23.4%)	36 (27.5%)	NS
Awake bruxism	43 (27.9%)	53 (40.5%)	NS

NS, nonsignificant; *p*^, level of significance after Bonferroni correction (Pearson Chi-Square and Fisher’s Exact Test were performed).

**Table 2:** Demographic comparison according to sex and time of examination.

	Total	Pre-COVID-19 (N = 108)	COVID-19 (N = 180)
Sex:			
Male	155 (53.8%)	59 (54.6%)	96 (53.3%)
Female	133 (46.2%)	49 (45.4%)	84 (46.67%)
Age (years):			
(Mean ± SD)	35.6 (12.4)	35 (11.45)	36.0 (12.92)
Median	32	31	32
Age (years):			
Male (Mean ± SD)	33.9 (11.00)	33.7 (10.28)	34.0 (18.81)
Female (Mean ± SD)	37.6 (13.58)	36.5 (12.55)	38.2 (14.11)

Pre-COVID-19, pre-pandemic era group; COVID-19, pandemic era group; SD, standard deviation.

**Table 3:** Sex comparison according to sex and time of examination.

	Men				Women			
	Pre-COVID-19	COVID-19	Total	*p*^	Pre-COVID-19	COVID-19	Total	*p*^
Parafunction	17 (28.8%)	61 (63.5%)	78 (50.3%)	<0.001	12 (24.5%)	50 (59.5%)	62 (46.6%)	<0.001
Pain in jaw function	1 (1.7%)	14 (14.7%)	15 (9.7%)	NS	8 (16.3%)	18 (21.4%)	26 (19.5%)	NS
Pain on awakening	3 (5.2%)	14 (14.7%)	17 (11.1%)	NS	3 (6.1%)	13 (15.5%)	16 (12%)	NS
Reported TMD pain	10 (16.9%)	17 (17.9%)	27 (17.5%)	NS	19 (38.8%)	22 (26.2%)	41 (30.8%)	NS
Pain on Temporal area	7 (12.1%)	18 (18.9%)	25 (16.3%)	NS	11 (22.4%)	21 (25%)	32 (24.1%)	NS
Sleep bruxism	10 (16.9%)	26 (27.4%)	36 (23.4%)	NS	6 (12.2%)	30 (36.6%)	36 (27.5%)	0.014
Awake bruxism	10 (16.9%)	33 (34.7%)	43 (27.9%)	NS	7 (14.3%)	46 (56.1%)	53 (40.5%)	<0.001

NS, nonsignificant; Pre-COVID-19, pre-pandemic era group; COVID-19, pandemic era group; *p*^, level of significance after Bonferroni correction (Pearson Chi-Square and Fisher’s Exact Test were performed).

**Table 4:** Sleep and awake bruxism according to sex and time of examination.

		No BRX	AB	SB	AB + SB	*p* *
Men	Pre-COVID-19	43 (72.9%)	6 (10.2%)	6 (10.2%)	4 (6.8%)	0.036
	COVID-19	47 (49.5%)	22(23.2%)	15 (15.8%)	11 (11.6%)	
	Total	90 (58.4%)	28(18.2%)	21 (13.6%)	15 (9.7%)	
Women	Pre-COVID-19	39 (79.6%)	4 (8.2%)	3 (6.1%)	3 (6.1%)	<0.001
	COVID-19	26 (31.7%)	26(31.7%)	10 (12.2%)	20 (24.4%)	
	Total	65 (49.6%)	30 (22.9%)	13 (9.9%)	23 (17.6%)	

Pre-COVID-19, pre-pandemic era group; COVID-19, pandemic era group; *p*, level of significance; * Pearson Chi-Square; No BRX, no bruxism; SB, sleep Bruxism; AB, awake bruxism.

## Data Availability

The data that support the findings of this study are available on request from the corresponding author. The data are not publicly available due to privacy or ethical restrictions.
